# Strategies to encourage freestall use in dairy heifers

**DOI:** 10.3168/jds.2021-0118

**Published:** 2021-10-09

**Authors:** Jennifer M.C. Van Os, Geoffrey S.S. Nemeth, Daniel M. Weary, Marina A.G. von Keyserlingk

**Affiliations:** 1 Animal Welfare Program, Faculty of Land and Food Systems, University of British Columbia, Canada V6T 1Z4; 2 Department of Animal and Dairy Sciences, University of Wisconsin-Madison, 1675 Observatory Dr., Madison 53706

## Abstract

Dairy cattle can experience problems adjusting to freestalls when first introduced, resulting in a marked reduction in lying time and increased abnormal behaviors such as lying in the alley or lying backward in a stall. Our objective was to evaluate 2 strategies to ease the transition of heifers to freestalls: using an older, experienced heifer as a social model [experiment (Exp.) 1] or using brushes mounted in the stalls as an attractant (Exp. 2). In Exp. 1, 44 naïve heifers (129 ± 37 d of age, mean ± standard deviation) were assigned in pairs (n = 11 pairs/treatment) to either the control or social model (with an older heifer, 200 ± 24 d of age, as an experienced social companion) treatments. In Exp. 2, 52 naïve heifers (146 ± 9 d of age) were preassigned in pairs (n = 13 pairs/treatment) to either control or brush treatments. In both studies, heifers were initially housed in pens with a bedded pack (for 5 and 7 d in Exp. 1 and 2, respectively) before moving to pens in the same barn with sand-bedded freestalls. On d −2, 0, and 4 relative to the move to the freestalls, standing, perching (with the front hooves on the bedding and rear hooves in the alley), and lying (location: bedding vs. alley; direction in stall on d 0 and 4: forward or backward) were recorded at 5-min intervals. In addition, starting on d 0 relative to the move, latency to first lie down in a stall was recorded continuously. In both studies, time budgets did not differ between treatments. On the day heifers were moved, they spent less time lying relative to d −2 [Exp. 1: −1.8 h/d, standard error (SE): 0.4 h/d; Exp. 2: −3.0 h/d, SE: 0.3 h/d] and more time standing and perching. By d 4, lying and standing returned to d −2 baseline levels, although heifers continued to spend more time perching. When heifers moved to freestalls, no treatment differences were observed in the latency to lie down in a stall, but latencies differed between Exp. 1 (averaging approximately 3.8 h across treatments) and Exp. 2 (averaging 31.4 h across treatments). After moving to freestalls, the percentage of total lying time that heifers spent facing forward in a stall increased (Exp. 2: 53 vs. 77%, SE: 7%) or tended to (Exp. 1: 84 vs. 92%, SE: 3%) between d 0 and 4 of exposure, with no effect of treatment. Although lying behavior improved with days of exposure to freestalls, some heifers continued to lie down in the alley or backward in a stall, suggesting the need for additional work to ease the adjustment to freestall housing.

## Highlights

• Transitioning to freestalls can be challenging for heifers.
• We evaluated the effects of social models and brushes on freestall use. No treatment effects were detected.
• Lying behavior improved with days of exposure to freestalls.
• Some abnormal behavior (lying in alley, lying backward in stall) persisted.

## Graphical Abstract

**Figure:**
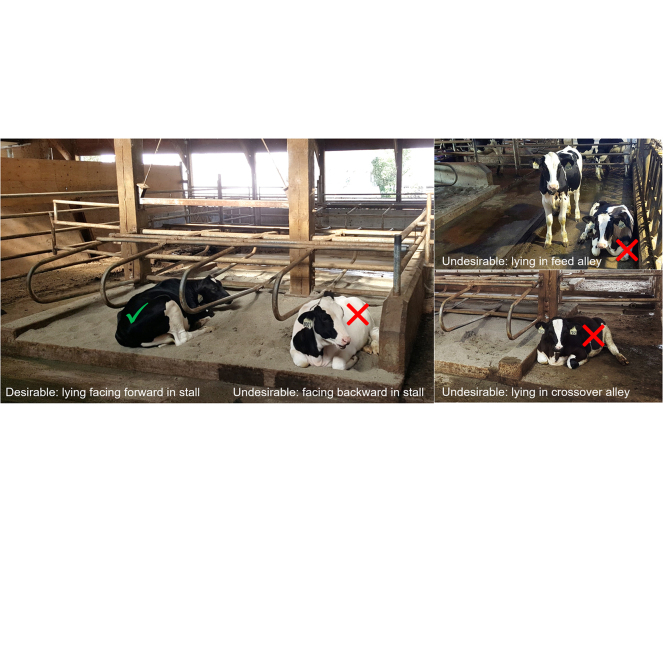
**Summary:** Across 2 studies, we evaluated novel strategies to ease the transition of naïve heifers to freestalls: using an older, experienced heifer as a social model or using stationary brushes mounted in the stalls as an attractant. In both studies, heifers were initially housed in bedded-pack pens before moving to pens with sand-bedded freestalls. Neither experimental manipulation resulted in detectable effects on freestall use, but lying behavior improved with days of exposure to freestalls. Nonetheless, some heifers continued to lie down in the alley or backward in a stall, suggesting the need for additional work to ease the adjustment to freestall housing.

Approximately 40, 30, and 13% of US dairy farms use freestalls as the main housing type for their lactating cows, dry cows, and weaned heifers, respectively (USDA, 2016). In this type of housing, the lying area is defined by hardware to separate and orient cattle (Ruud and Bøe, 2011). This positioning is intended to encourage cattle to eliminate into an alleyway and thus avoid soiling the lying surface, keeping cattle clean and reducing fecal contamination of the udder that can increase mastitis risk (Kjæstad and Simensen, 2001; Schreiner and Ruegg, 2003). Stalls may also help keep bedding clean and dry, reducing bedding and labor costs. The USDA reported a lower proportion of dirty cows in freestalls compared with dry lot systems (10 vs. 22%, respectively; USDA, 2010).

Although freestalls have benefits, naïve cattle can experience problems in initially adjusting to them, resulting in a marked reduction in lying time when first introduced (von Keyserlingk et al., 2011) and undesirable behaviors such as lying in the alley (O'Connell et al., 1993; Kjæstad and Myren, 2001b; von Keyserlingk et al., 2011) or lying backward in a stall. Perching, defined as standing with the front feet in the stall and the back feet in the alley, is another undesirable behavior in freestalls because it is a risk factor for lameness in adult cattle (Bernardi et al., 2009).

Little research has examined the adjustment of dairy cattle to freestall housing or how this might be improved. One study attempted to make stalls more attractive by covering the stall base with mats and by providing feed in the stalls (O'Connell et al., 1993). Dairy cows are motivated to use brushes (McConnachie et al., 2018), and younger heifers are known to use stationary brushes (Pempek et al., 2017; Horvath et al., 2020), particularly with their heads (Van Os et al., 2021). Therefore, we hypothesized that adding brushes inside the front of the stalls might encourage proper stall use. Another untested hypothesis was that older, experienced animals could serve as social models to encourage stall use. In previous work, the presence of older, experienced conspecifics increased the grazing activity of inexperienced dairy heifers (Costa et al., 2016) and beef steers (Hessle, 2009), as well as the diversity of plants and shrubs consumed by heifers on unfamiliar grazing sites (Velázquez-Martínez et al., 2010). Similarly, the presence of older, experienced companions increased feeder visits and time spent consuming solid feed by preweaning dairy heifers (de Paula Vieira et al., 2012).

In the current study, we assessed 2 interventions: in experiment (**Exp.**) 1, we assessed the use of an older, experienced animal as a social model, and in Exp. 2, we assessed the use of stationary brushes mounted inside the stalls as attractants. In both experiments, the objectives were to evaluate effects on stall use and time budgets before and after naïve heifers moved to freestall housing. We hypothesized that the presence of older, experienced heifers and brushes in the stalls would both increase stall use.

The experiments were conducted between June and November 2016 (Exp. 1) and June and October 2017 (Exp. 2) at The University of British Columbia (UBC) Dairy Education and Research Centre (Agassiz, BC, Canada). All procedures were approved by the UBC Animal Care Committee (protocol A14-0245). The sample sizes in both studies were based on a power analysis using the changes in lying time reported in von Keyserlingk et al. (2011); with large effect sizes (Cohen's d ≥0.8) expected, a sample size of n = 10 to 13 experimental units was required to achieve a power of 0.8 or higher.

In Exp. 1, 44 naïve Holstein heifers were enrolled at (mean ± SD) 129 ± 37 d of age. Eleven older heifers (200 ± 24 d of age) served as experienced social companions. These older heifers were selected based on 3 criteria: (1) they had lived in freestall housing for >2 mo, (2) they were 1 to 3 mo older than the naïve heifers, and (3) they were not observed lying in the alley, lying backward in a stall, or displacing other heifers from stalls during 72 h of continuous video observation in freestall housing. Pairs of naïve heifers were assigned pseudo-randomly to either the social model or control treatment (n = 11 pairs/treatment), balancing for age (social model vs. control treatment: 130 ± 38 vs. 127 ± 38 d of age, respectively, mean ± SD). In the social model treatment, each pair of naïve heifers was housed with 1 older, experienced heifer. In the control treatment, the pair of naïve heifers were the only animals in the pen. To maintain the same number of naïve heifers per treatment, the total number of animals per pen differed. Nonetheless, this confounder meant that in the second phase of the study, fewer stalls would be available per animal in the social model treatment, thus reducing the potential to bias the results in favor of our hypothesis.

In the first phase of the study, heifers were housed in bedded-pack experimental pens for 5 d beginning at 0900 h, when naïve heifers in the social model treatment were first introduced to the older, experienced heifers. Two adjacent, mirror-image pens were used, with treatments balanced between the pens. The pens were enclosed on 3 sides with 1.8-m-high plywood to prevent visual and physical contact between treatments. The 4.6- × 7.1-m lying area was bedded with 12 cm of sawdust and separated from the feeding area by a 4.8- × 3.1-m concrete alley covered with texturized rubber. The feed barrier had nonmoving bars slanted at a 60° angle (13 feeding spaces with a center-to-center distance of 0.3 m between bars). Heifers had ad libitum access to long-stemmed fescue grass hay top-dressed with texturized calf grower (Hi-Pro Feeds Ltd.). The previous day's refusals were removed and fresh feed delivered at approximately 0900 h, and the feed was pushed up at approximately 1500 and 2200 h. Heifers had ad libitum access to fresh drinking water from a self-filling trough on a raised platform accessible from the alley. The feed alley was cleaned by an automatic manure scraper 6 times/d, and the lying area was cleaned manually during feeding at 0900 h. Fresh sawdust bedding was added between groups.

In the second phase of the study, on the sixth day (d 0 relative to the move) between 0900 and 1100 h, each group of heifers was moved to 1 of 2 adjacent freestall pens within the same barn, enclosed on 3 sides with plywood. No other nonexperimental heifers were housed in these pens. Until this point, the naïve heifers had no exposure to freestalls or feed barriers with self-locking headlocks. Each 6.7- × 12.2-m pen had 13 freestalls in 3 rows of 4, 4, and 5 stalls (from the feed bunk to the back of the pen); the back 2 rows were separated by a 6.7- × 3.1-m alley. Adjacent to the first row of freestalls was a 1.3- × 1.9-m raised crossover alley. The freestalls were deep bedded with sand and measured 1.8 m long, with 0.9 m center-to-center distance between freestall loops, and had 2 neck rails (1.2 and 1.4 m away from the curb, 0.8 m high from the stall base to the bottom of the rail). The 6.7- × 3.1-m feed alley had 13 feeding spaces with headlocks (0.4 m center-to-center distance). Heifers were fed the same diet on the same schedule as in the bedded-pack phase. Water was available ad libitum from an automatically filled trough on a raised platform adjacent to the stalls. All alleys were covered with texturized rubber. The pens were scraped automatically 6 times/d, and the stalls were raked manually once daily during feeding at 0900 h. Heifers were removed from the experimental pen on d 5, relative to the move to freestalls. Between groups, the stalls were topped off with fresh sand and raked.

In both phases of the study, behavior was recorded using 2 video cameras/pen (Panasonic WV-CP214 24V) mounted 8 m above each pen along with a 100-W red light to aid nighttime visibility. One observer (G.N.) scored behavior in Excel (Microsoft Corp.) spreadsheets for all heifers at 5-min intervals on d −2, 0, and 4 relative to the move to the freestall pens. The behaviors recorded were posture (lying, standing, or perching with the front hooves on the bedding and rear hooves in the alley) and eating (a subset of standing, with the head through the feed barrier or headlocks and lowered to the ground). Within lying, the location (bedding vs. alley) was recorded, and on d 0 and 4 in the freestall pens, the direction heifers were facing (forward vs. backward) when lying in a stall was also recorded. In addition, the same observer watched video continuously when heifers moved to the freestall pens to record their latencies to first lie down anywhere in the pen (and the location, alley vs. stall), first lie down in a stall (and the direction, forward vs. backward), and to first eat with their heads through the headlocks.

In Exp. 2, 52 naïve heifers were enrolled at a mean (± SD) of 146 (± 9) d of age. In the first phase of the study, heifers were housed in groups of 4 (n = 13 groups) for 7 d in a single bedded-pack pen, enclosed on 3 sides with plywood. Groups entered the pen sequentially based on birthdate, which kept age relatively consistent among groups. Housing and management were identical to the first phase of Exp. 1, with 2 exceptions: (1) for the requirements of another study (Van Os et al., 2021), the lying area was not cleaned during data collection, but the area surrounding the water trough was scraped manually during feeding at 0900 h, and (2) feed was pushed up at approximately 1100, 1500, 1900, and 2200 h. To familiarize heifers with brushes for the second phase of the study, 4 scrub brushes (Pro Series 10” Wash Brush, 25.4 cm-long × 6.0 cm-wide with 3.8-cm-long bristles; Camco Brush) were mounted on the fence surrounding the bedded area. Behavior was recorded using 3 video cameras mounted above the pen along with a red light, as in Exp. 1.

On the eighth day (d 0 relative to the move) at 1030 h, each group of heifers was divided into preassigned treatment pairs and moved to 1 of 2 freestall pens on opposite sides of a 6.3-m-wide center alley within the same barn. Until this point, all heifers were naïve to freestalls and feed bunks with self-locking headlocks. Pairs of heifers had been preassigned pseudo-randomly to either the brush or control treatment (n = 13 pairs/treatment) before phase 1 of the study, balancing for pen location and age (brush vs. control: 154 ± 10 vs. 153 ± 9 d of age at the start of phase 2, mean ± SD). In the brush treatment, each freestall had a brush mounted horizontally on the rail 0.2 m above the stall base in the front of the stall. In the control treatment, the pen contained no brushes. Each experimental pen had access to only the first row of 4 freestalls facing away from the feeding area. The back of the crossover alley adjacent to the freestalls was blocked with a gate to prevent heifers from accessing the rest of the pen, which remained empty. Heifers were fed a TMR, available ad libitum and delivered and pushed up on the same schedule as in the bedded-pack phase. The pens were scraped automatically 6 times/d, and the stalls were raked and the area around the water trough was scraped manually during feeding at 0900 h. Pairs of heifers were removed from the pen at approximately 1100 h on d 6, relative to the move to the freestalls. Between pairs, the stalls were topped off with fresh sand and raked. A video camera and a red light were mounted 8 m above each pen. One observer (J.V.) scored behavior for all heifers in 5-min intervals on d −2, 0, and 4, relative to the move to the freestall pens. The behaviors were the same as in Exp. 1, except that eating was not recorded. The same observer watched video continuously when heifers moved to the freestall pens, recording latencies as in Exp. 1, with the exception of feeding behavior, and with the addition of the latency for heifers to first enter a stall with at least 1 hoof.

All analysis was conducted using SAS (version 9.4; SAS Institute, 2011). Statistical comparisons were conducted using pairs of naïve heifers as the experimental unit; data were summarized as the pair mean before analysis. Significance was declared at *P* < 0.05 and tendencies at *P* < 0.1. Linear mixed models were constructed using PROC MIXED. The assumption of equal variance between treatments was assessed graphically using boxplots (PROC UNIVARIATE). The assumption of normality was evaluated by plotting residuals (PROC PLOT), and when data were not normally distributed (i.e., latencies), natural log transformations were applied (Martin and Bateson, 2007). Although these transformations normalized residuals, inferences were unchanged (i.e., significant values remained so and vice versa), so we retained data in the original scale for ease of interpretation.

When naïve heifers moved to the freestall pens, their latency to first feed through the headlocks (Exp. 1 only), enter a stall with at least one hoof (Exp. 2 only), lie down anywhere in the pen, and lie down in a stall were evaluated using mixed models with a fixed term for treatment (Exp. 1: social model vs. control; Exp. 2: brush vs. control) and a random term for pair of heifers, nested within treatment. We predicted that heifers in the social model (Exp. 1) and brush (Exp. 2) treatments would feed through the headlocks (Exp. 1), enter a stall (Exp. 2), and lie down in a stall (both studies) sooner than those in the control treatments. In addition, the proportion of heifers choosing a stall (vs. the alley) as their first lying location and the proportion facing forward (vs. backward) the first time they lay down in a stall are reported descriptively.

Not all heifers were observed performing undesirable lying behaviors. Descriptive data for lying in the alley (d −2, 0, and 4) and lying in a stall backward (d 0 and 4) are reported, along with the proportion of heifers showing only desirable lying behaviors (i.e., lying in a stall facing forward). The percentage of lying time spent lying in a stall facing forward was calculated on each day and evaluated using a mixed model with fixed terms for treatment (Exp. 1: social model vs. control; Exp. 2: brush vs. control), day (0 and 4), and the treatment × day interaction, with a random term for pair of heifers, nested within treatment. We predicted that heifers in the social model (Exp. 1) and brush (Exp. 2) treatments would spend a greater percentage of their total lying time in a stall facing forward relative to those in the control treatments, and that this percentage would increase with day of exposure to the freestalls.

Finally, overall time budgets were evaluated. In Exp. 1, the amounts of time that inexperienced heifers spent lying, standing, eating (a subset of standing), and perching were evaluated using mixed models with fixed terms for treatment (social model vs. control), day (−2, 0, and 4 relative to the move to freestalls), and treatment × day interaction, with a random term for pair of heifers, nested within treatment. In Exp. 2, the amounts of time heifers spent lying, standing, and perching were evaluated using mixed models with fixed terms for treatment (brush vs. control), day (−2, 0, and 4 relative to the move to freestalls), and the treatment × day interaction, with a random term for pair of heifers, nested within treatment. For both studies, where significant effects of day were found, contrasts between days were conducted using the PDIFF function. We predicted that lying and eating time (Exp. 1 only) would be reduced on d 0 relative to d −2 and 4 but that heifers in the social model and brush treatments would show less reduction relative to those in the control treatments.

In Exp. 1, before moving to the freestalls (d −2), few naïve heifers were observed lying in the bedded-pack pen alleys (0 vs. 3 heifers in the social model vs. control treatment, for a maximum of 1.1 h in the latter). Upon moving, there was no effect of treatment on the latency to first feed through the headlocks, lie down anywhere in the pen, or lie down in a stall (Table 1; *F*_1,20_ ≤ 0.6, *P* ≥ 0.45). Most heifers chose a stall (vs. the alley) for their first lying location (73 vs. 68% of heifers in the social model vs. control treatment), and 86 versus 91% of heifers faced forward when they first lay down in a stall.

**Table 1:** Treatment differences in the latency for heifers to show various behaviors upon moving from a bedded-pack pen to a freestall pen in 2 studies, along with the percentage of total lying time spent facing forward in a stall on d 0 and 4 relative to the move

Behavior	Exp. 1^1^			Exp. 2^2^		
	Social model	Control	SE	Brush	Control	SE
Latency to feed through headlocks^3^	0.3 h	0.2 h	0.1 h	—	—	—
Latency to enter a stall with at least one hoof^4^	—	—	—	2.5 min	6.2 min	2.3 min
Latency to lie down anywhere in pen	2.1 h	3.0 h	0.9 h	2.5 h	2.5 h	0.2 h
Latency to lie down in a stall	3.2 h	4.5 h	1.5 h	31.3 h	31.5 h	13.5 h
Percentage of total lying time facing forward in a stall						
d 0	86%	82%	5%	55%	50%	10%
d 4	97%	86%	5%	80%	74%	10%

1 Experiment (Exp.) 1: Using an older, experienced heifer as a social model.

2 Exp. 2: Using brushes mounted in the stalls as an attractant.

3 Recorded only in Exp. 1.

4 Recorded only in Exp. 2.

However, on d 0 in the freestalls, only 27 versus 32% of heifers in the social model versus control treatments showed only desirable lying behavior (i.e., lying only in a stall facing forward), with 50 versus 64% observed lying in the alley and 36 versus 32% lying backward in a stall. By d 4, 82 versus 55% of heifers in the social model versus control treatments showed only desirable lying behavior, with only 9 versus 23% lying in the alley and 14 versus 27% lying backward in a stall.

Heifers spent most of their total lying time facing forward in a stall, regardless of treatment (Table 1; no treatment × day interaction, *F*_1,20_ ≤ 2.1; *P* ≥ 0.16). There was a tendency for a day effect: the percentage of lying time facing forward increased from d 0 to 4 (84 vs. 92%, SE: 3%; *F*_1,20_ = 3.2; *P* = 0.089). This difference was driven by a reduction in time spent lying in the alley; heifers in both treatments spent 1.2 versus 0.3 h (SE: 0.3 h) lying in the alley on d 0 versus 4. When lying in a stall, heifers in both treatments were observed lying backward for 0.7 versus 1.0 h (SE: 0.3 h) on d 0 versus 4.

There was no effect of treatment on time budgets (*F*_1,20_ ≤ 0.6, *P* ≥ 0.45; no treatment × day interactions, *F*_2,40_ ≤ 0.5, *P* ≥ 0.59), but there were effects of day (*F*_2,40_ ≥ 4.4, *P* ≤ 0.019; Figure 1A). Heifers spent less time lying (*P* < 0.0001) and eating (*P* < 0.020) on the day they moved to freestalls (d 0) relative to d −2 or d 4; by d 4, durations for both behaviors were equivalent to baseline values on d −2 (*P* ≥ 0.49). On d 0, heifers spent more time perching and standing than on d −2 or 4 (*P* < 0.001); on d 4, durations of perching remained greater than on d −2 (*P* = 0.011), whereas standing was equivalent between d −2 and 4 (*P* = 0.11).

**Figure 1:**
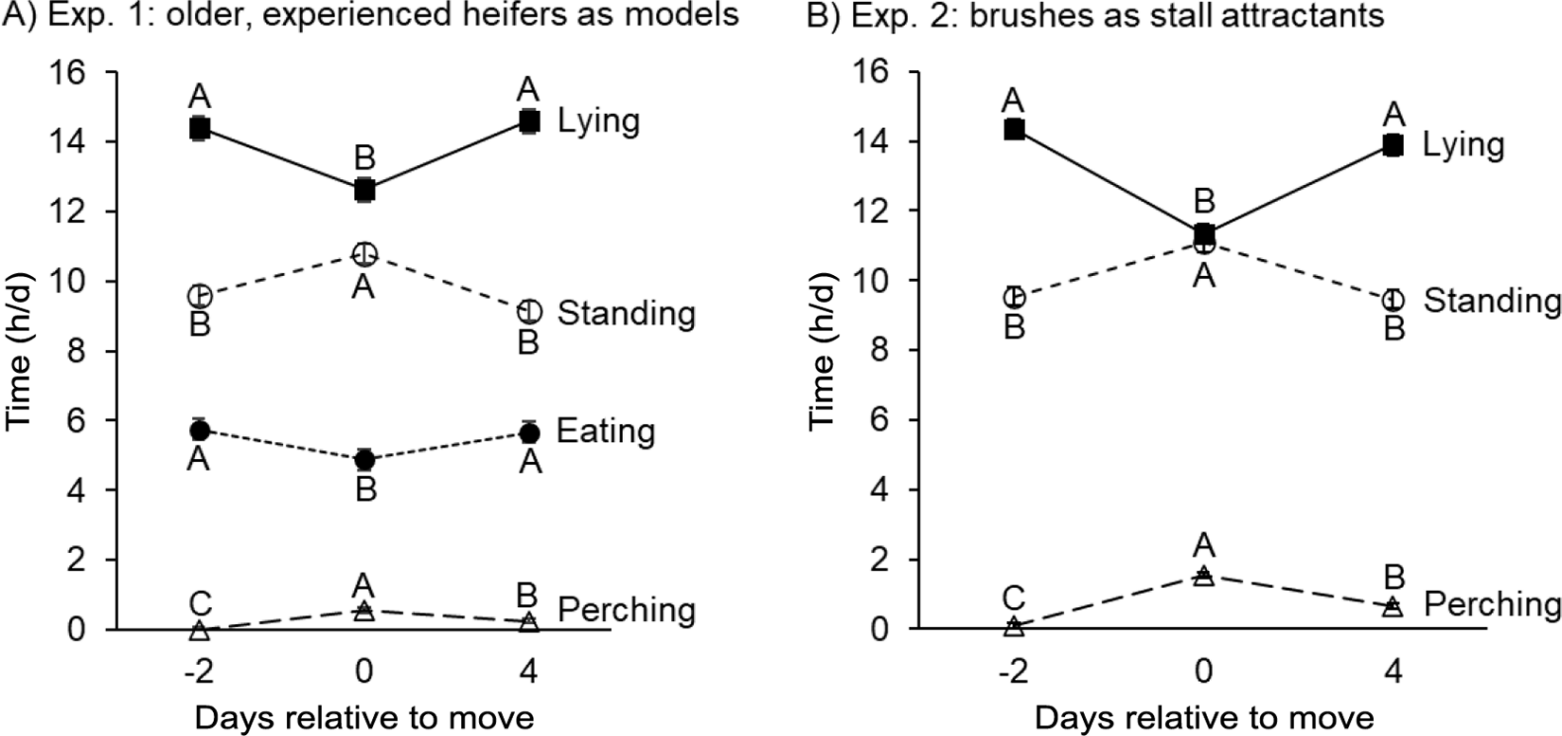
The amount of time (h/d; mean ± SE) heifers spent lying, perching, standing, and eating [a subset of standing; experiment (Exp.) 1 only] on d −2, 0, and 4, relative to their move from a bedded-pack pen to a freestall pen in (A) Exp. 1, and (B) Exp 2. Letters (A–C) indicate significant (*P* < 0.05) differences between days for a given behavior.

All older, experienced heifers chose a stall for their first lying location on d 0, and all lay facing forward in the stall. Only desirable lying behavior was shown on d 0 by all but 2 of these heifers (who spent 1.8 and 3.1 h lying backward in a stall); one of these heifers spent 0.2 h lying backward in a stall on d 4. No lying in the alley was observed on any day. The older, experienced heifers spent 13.7, 13.8, and 14.1 h lying on d −2, 0, and 4 (SE: 0.5 h/d). They spent 0.0, 0.4, and 0.3 h perching (SE: 0.1 h/d); 10.3, 9.8, and 9.6 h standing (SE: 0.5 h/d); and 6.0, 4.9, and 5.6 h eating (a subset of standing; SE: 0.5 h/d) on those days.

In Exp. 2, before moving to the freestalls (d −2), only one heifer (assigned to the control treatment) was observed lying in the alley of the bedded-pack pen (for 0.6 h). Upon moving heifers to the freestall pen, there were no treatment differences in latency to first enter a stall with at least one hoof or lie down anywhere in the pen (Table 1; *F*_1,24_ ≤ 1.3, *P* ≥ 0.26). Few heifers chose a stall (vs. the alley) for their first lying location (15 vs. 31% in the brush vs. control treatment) or showed only desirable lying behavior on d 0 (4 vs. 22%); 92 versus 78% were observed lying in the alley, and 13% in each treatment were observed lying backward in a stall.

On average, heifers took more than 1 d to first lie down in a stall, regardless of treatment (Table 1; *F*_1,22_ = 0.0, *P* = 0.99). When heifers first lay down in a stall, the majority faced forward (90 vs. 85%). By d 4, 69 versus 54% of heifers in the brush versus control treatment showed only desirable lying behavior, with 23 versus 42% lying in the alley and only 8 versus 12% lying backward in a stall. Heifers spent most of their lying time facing forward in a stall, regardless of treatment (Table 1; no treatment × day interaction, *F*_1,22_ = 0.0; *P* = 0.98). There was a day effect: the percentage of lying time facing forward in a stall increased from d 0 to 4 (53 vs. 77%, SE: 7%; *F*_1,22_ = 18.2; *P* < 0.001). This difference was driven by a reduction in time spent lying in the alley; heifers in both treatments spent 4.6 versus 2.6 h (SE: 0.8 h) lying in the alley on d 0 versus 4. When lying in a stall, heifers in both treatments spent on average 0.4 h (SE: 0.2 h) lying backward on d 0 and 4.

There was no effect of treatment on time budgets (*F*_1,24_ ≤ 0.1, *P* ≥ 0.77; no treatment × day interaction, *F*_2,46_ ≤ 1.4, *P* ≥ 0.26), but behaviors varied with day (*F*_2,46_ ≥ 21.1, *P* < 0.0001; Figure 1B). On the day heifers were moved to the freestalls (d 0), they spent less time lying and more time standing and perching relative to d −2 or 4 (*P* < 0.0001). By d 4 in the freestalls, both lying and standing returned to d −2 baseline levels (*P* ≥ 0.11), although heifers continued to spend more time perching on d 4 versus d −2 (*P* < 0.001).

In both experiments, we evaluated strategies for improving the transition of naïve heifers to freestalls: older, experienced heifers as social models and brushes mounted in the freestalls as attractants. In neither study were differences detected between the experimental and control treatments for any of the key outcome measures, including latency to first lie down in a stall, the percentage of total lying time spent facing forward in a stall, and the duration of lying (vs. perching or standing) when heifers moved to freestalls. In Exp. 1, the naïve heifers became familiar with the social models before moving to the freestalls. However, we did not quantify lying proximity during either phase of the study; future work could examine the extent to which younger heifers avoid or choose to lie near older heifers. In Exp. 2, although the heifers used the brushes, they did so for only 4 min, on average, on the day they moved to the freestalls (Van Os et al., 2021). The combination of environmental changes between the bedded-pack and freestall pens, including the bedding, feed (Exp. 2), feed barrier, and stall hardware (i.e., freestall partitions and neck rails), might have overwhelmed any detectable effects of the social models or brushes. Future research could also examine other strategies. For example, some commercial dairy operations have reported success using a stepwise approach by introducing new elements (e.g., pen hardware, bedding, feed types, feed and water sources) separately instead of at once.

Interesting descriptive similarities and differences emerged between the 2 experiments. In both, the latency for heifers to first lie down anywhere in the freestall pen was similar (approximately 2–3 h), but there was a marked difference in the latency to first lie down in a stall (on average, 3.8 h in Exp. 1 vs. 31.4 h in Exp. 2). Similarly, over two-thirds of heifers in Exp. 1 chose a stall as their first lying location, but less than one-third did so in Exp. 2. These differences cannot be easily explained, as the studies were conducted in the same research facility, in a similar season, with inexperienced heifers around the same age (4–5 mo). Stocking density was lower in Exp. 1, in which heifers had access to all 13 stalls in the pen (6.5 vs. 4.3 stalls/heifer in the social model vs. control treatments, respectively) versus only 4 stalls (2 stalls/heifer) in Exp. 2. However, the implication of this difference is unclear because heifers in both studies had access to more than one stall, whereas ≤1 stall is provided per animal on typical commercial dairy farms.

In both experiments, lying behavior improved with days of exposure to the freestalls, as characterized by lying duration and the percentage of lying time spent facing forward in the stall. In 2 previous studies, heifers of a similar age showed a marked reduction in lying time when first introduced to freestalls (by 3 vs. 4 h/d for heifers 159 ± 25 and 162 ± 26 d of age, respectively; von Keyserlingk et al., 2011). In the present study, the magnitude of the reduction in lying time was nearly identical in Exp. 2 (3 h/d) but slightly less in Exp. 1 (1.8 h/d, on average). Both lying and standing (either in the alleys or fully in the bedding) returned to baseline by d 4 of exposure to freestalls, but perching remained higher than at baseline. Although perching is commonly observed, it is considered undesirable because it is a behavioral risk factor for lameness in adult cows (Bernardi et al., 2009).

In the bedded-pack pens, heifers were rarely observed lying in the alley. Across days of exposure to freestalls, heifers spent more time facing forward in a stall, representing over three-fourths of total lying time on d 4. The number of heifers showing only desirable lying behavior increased with time (fewer than one-third vs. more than half on d 0 vs. 4). On the day heifers moved to freestalls, more than half in both Exp. 1 and 2 were observed lying in the alley; by d 4 a minority of heifers were observed still doing so. Previous work reported an increase in lying in the alley when cattle were initially moved to freestall housing (O'Connell et al., 1993; Kjæstad and Myren, 2001b; von Keyserlingk et al., 2011). This problem can persist with age: 54% of Norwegian dairy producers reported having cows who refused to lie in the stalls (out of 184 respondents; Kjæstad and Myren, 2001a). Lying backward in a stall, which can result in soiling the stall, was previously reported by von Keyserlingk et al. (2011) in this research facility and has been observed by the authors on commercial dairy farms. In the present study, >85% of heifers faced forward the first time they lay down in a stall. Interestingly, the number of heifers lying backward in a stall decreased by d 4 of exposure in Exp. 1, but the opposite pattern was observed in Exp. 2. Future research could elucidate the individual factors contributing to the persistence of this behavior.
